# Tobacco Treatment Guideline for High Risk Groups: A pilot study in patients with Chronic Obstructive Pulmonary Disease

**DOI:** 10.18332/tid/85944

**Published:** 2018-04-12

**Authors:** Antigona C. Trofor, Sophia Papadakis, Lucia Lotrean, Ioana Buculei-Porosnicu, Vergina K. Vyzikidou, Vaso Evangelopoulou, Constantine Vardavas, Panagiotis Behrakis

**Affiliations:** 1 University of Medicine and Pharmacy Grigore T. Popa, Iasi, Romania; 2 Division of Prevention and Rehabilitation, University of Ottawa Heart Institute, Ottawa, Canada; 3 Faculty of Medicine, University of Ottawa, Ottawa, Canada; 4 Iuliu Hatieganu University of Medicine and Pharmacy, Cluj-Napoca, Romania; 5 Clinical Hospital of Pulmonary Diseases, Iasi, Romania; 6 George D. Behrakis Research Lab, Hellenic Cancer Society, Athens, Greece; 7 Hellenic Centre for Disease Control and Prevention, Athens, Greece; 8 Institute of Public Health, American College of Greece, Athens, Greece; 9 Biomedical Research Foundation of the Academy of Athens (BRFAA), Athens, Greece

**Keywords:** tobacco treatment, guidelines, Chronic Obstructive Pulmonary Disease, cessation

## Abstract

**INTRODUCTION:**

Smoking cessation is a key clinical intervention for reducing progressive lung destruction and lung function deterioration in patients with Chronic Obstructive Pulmonary Disease (COPD). Specialised Tobacco Cessation Guidelines for High-risk Groups (TOB-G) were developed and published in 2017 that present evidence-based recommendations to support smoking cessation in COPD patients. The purpose of this pilot study was to examine the real world effectiveness of the TOB-G guideline recommendations among a sample of COPD patients.

**METHODS:**

A pilot study was conducted among a sample of COPD patients who smoke and were interested in quitting. Participants were recruited from inpatient and outpatient hospital admissions between October and December 2016 in Iasi, Romania. The intervention program was designed based on the recommendations of the TOB-G guidelines for COPD patients. Patients received a total of four contacts: at baseline, 1, 2, and 6 months. The primary outcome measure was biochemically validated point prevalence smoking abstinence measured at 6 months.

**RESULTS:**

Fifty patients (74% male; age mean±SD = 60.2±7.8) with diagnosed COPD took part in the pilot study. Self-reported rates of point prevalence smoking abstinence were 30.6%, 44.9% and 64.6% at the 1-, 2-, and 6-month follow-up, respectively. Carbon monoxide testing was completed with 51.6% of the sample at 6 months. The biochemically verified abstinent rate was 33.3% at the 6-month follow-up.

**CONCLUSIONS:**

This pilot testing of the TOB-G Clinical Practice Guidelines for COPD patients was associated with high rates of patient smoking abstinence, which are of clinical importance. Further research is needed to evaluate the guidelines large-scale effectiveness in clinical practice.

## INTRODUCTION

Tobacco use is associated with increased mortality, deterioration of pulmonary function, including an early age decline of forced expiratory volume (FEV_1_), and an accelerated annual decline of FEV_1_^1-3^.

Despite the known risks of continued tobacco use on disease outcomes, an estimated 33-35% of patients with Chronic Obstructive Pulmonary Disease (COPD) continue to smoke^4-6^. The prevalence of tobacco use among patients with COPD has been shown to vary based on disease severity with the rate being 54% to 77% in mild COPD patients, and 38% to 51% in those with severe COPD^6^, hence patients with COPD who smoke constitute a high-risk population.

Smoking cessation is the single most effective treatment for reducing the rate of COPD progression and for improving disease outcomes^7,8^. Smoking cessation has been shown to improve both the prognosis^9^ and slow progression of the disease^10^, as well as reduce exacerbations of COPD and improve survival^3,11,12^. Respiratory symptoms (i.e. cough, sputum production, shortness of breath) and immune response have been shown to improve within 3-9 months of cessation, and lung function may increase by 10%^13,14^. Improved efficacy of therapies, including oxygen therapy and COPD inhalator medication such as bronchodilators^2^ or inhaled corticosteroids^15^ are expected after quitting.

There is emerging evidence that smokers with COPD have characteristics that may make it more difficult to quit smoking relative to the general population of tobacco users^4,16-18^. In particular, tobacco users with COPD have a history of smoking more cigarettes per day and are more highly addicted to nicotine compared to the general population of tobacco users^5,18^. COPD disease pattern that often includes the existence of co-morbidities (e.g. depression, anxiety, etc.) is associated with a lack of motivation and self-confidence for quitting, thus reducing the odds of quitting smoking^19,20^. Understanding these differences and tailoring cessation interventions may assist with increasing effectiveness of smoking cessation treatments among smokers with COPD^21^.

In 2017 within the context of the European Commission CHAFEA 3rd Health Programme, the Tobacco Cessation Guidelines for High-risk Groups (TOB-G) were developed with the aim to develop and implement an innovative and cost effective approach to prevent chronic diseases related to tobacco dependence by developing specialized tobacco cessation guidelines for populations at high risk^22,23^. The TOB-G guidelines include a special chapter on tobacco treatment among patients with COPD with 16 evidence-based clinical practice recommendations (Table 1). The guidelines identify intensive counselling and pharmacotherapy as the most effective cessation treatments for smokers with COPD^22^. The guidelines also suggest that treatment for smoking cessation in COPD patients should include motivational interviewing and personalized feedback^15,24^. Other non-pharmacological interventions, such as the use of COPD clinical questionnaires, spirometry ‘lung age’ testing, and biochemical validation of self-declared smoking status are being examined as adjunct strategies for increasing patient motivation to quit in this category of tobacco users^22,24-25^. However, further investigation is needed to explore COPD patients’ perspectives of obtaining their lung age to help motivate them to quit^15^.

**Table 1:** TOB-G Clinical Practice Guideline Recommendations for tobacco treatment in patients with COPD

*Recommendation*	*Level of Evidence*
1. Among COPD patients who continue to smoke, smoking cessation is the key clinical intervention for reducing progressive lung destruction and lung function deterioration and should be a clinical priority for all patients.	A
2. Co-habitants and families of COPD patients should be instructed not to expose COPD patients to tobacco smoke and should be included in smoking cessation programs.	D
3. All health care providers who treat COPD patients who smoke should be aware of the specific tobacco use and cessation patterns of this group of patients in order to tailor intervention strategies and increase success with quitting.	D
4. Smoking cessation interventions should be integrated into routine care of COPD patients who smoke, in both primary care and specialist settings.	A
5. Primary care providers, pulmonologists and other health professionals involved in the treatment of COPD should be trained in evidence-based smoking cessation treatment and be prepared to provide smoking cessation pharmacotherapy and counselling to their COPD patients or may refer them to a colleague trained in smoking cessation.	A
6. A combination of high-intensity counseling and pharmacotherapy is the most effective strategy for treating tobacco use in patients with COPD.	B
7. Exhaled air carbon monoxide (CO) and cotinine are useful non-invasive biomarkers of tobacco smoke exposure and can be used in clinical settings to assess smoking status and to monitor smoking cessation.	A
8. Clinicians overseeing the care of COPD smokers should take the opportunity to assess CO values whenever possible in follow-up visits and use it as a motivational tool to support quit attempts, being at the same time aware of the higher CO levels due to airway inflammatory process.	B
9. The role of ‘lung age’ for increasing patient motivation to quit smoking deserves further investigation.	C
10. A growing body of evidence suggests that majority of COPD patients, in particular those who report high levels of nicotine dependence will require a structured and intensive smoking cessation support in order to quit.	C
11. NRT can be used to support cessation among COPD patients; however standard dosing of NRT among COPD populations has produced lower quit rates than in the general population of smokers.	A
12. High dose NRT is recommended for COPD patients who report moderate to high levels of nicotine addiction as measured by the Fagerström Test of Nicotine Dependence. The combination of two types of NRT with different types of delivery is highly recommended.	A
13. Increasing the length of time that NRT is used to up to six or twelve months can be effective in increasing abstinence rates in COPD smokers compared to the standard 10 weeks of NRT therapy.	A
14. For COPD patients with low motivation to quit, NRT may be used to support gradual smoking reduction.	B
15. Varenicline is a first-line quit smoking medication that has been shown to be effective in supporting cessation in smokers with COPD, regardless of disease severity or number of cigarettes smoked.	B
16. Bupropion is an effective aid to support smoking cessation among COPD patients and it is safe to use bupropion in this population of tobacco users.	B

Understanding the real world effectiveness of guidelines is key in supporting their dissemination. As such, we conducted a pilot study to examine the effectiveness of a cessation intervention based on the TOB-G guideline recommendations on cessation rates among a sample of patients with COPD.

## METHODS

### Design

A single group non-randomized pilot study was conducted among a sample of COPD patients who smoke. Outcome measurement occurred at baseline, 1, 2, and 6 months. This study was approved by the Ethics Board of the Clinical Hospital of Pulmonary Diseases, Iasi, Romania (Protocol number 664292).

### Sample

**Figure 1:**
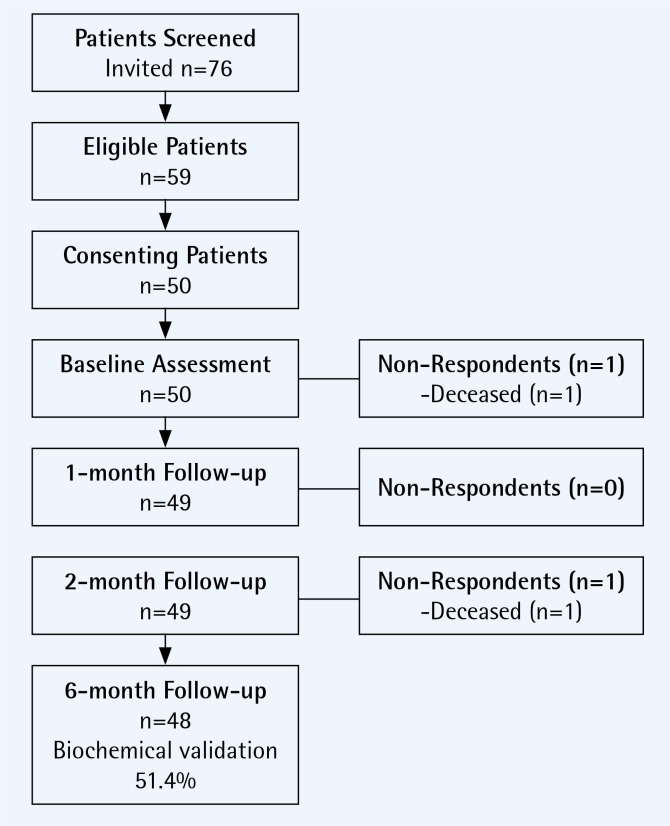
Recruitment flow

The study sample was recruited from inpatient and outpatient visits to the Clinical Hospital of Pulmonary Diseases in Iasi Romania between October and December 2016. All COPD patients had their smoking status assessed and received brief advice to quit smoking and were invited to enrol in the pilot study. Eligible patients were: daily tobacco users (>5 cigarettes per day) with a clinical diagnosis of COPD, who were interested in making a quit attempt and had access to a telephone for follow-up. The TOB-G guidelines address both inpatient and outpatient populations and as such the study design included for recruitment of both patient populations. All eligible patients provided written informed consent and were scheduled for an appointment in the outpatient pulmonology clinic of the hospital. Fifty eligible COPD patients participated in the pilot study. The recruitment flow diagram is presented as Figure 1. The primary reasons for non-participation was the distance and difficulty in traveling to the hospital to attend follow-up appointment (n=11) and preference to quit on one’s own or at a later date (n=6). Two patients died during the course of the study. Follow-up data were available for 100% of the remaining participants (n=48).

### Intervention

The intervention program was designed based on the evidence-based recommendations for tobacco treatment in the TOB-G clinical practice guidelines for COPD patients (Table 1). The intervention protocol was delivered based on the 5As (ask, advise, assess, assist, arrange) model for smoking cessation, which had been adapted for COPD patients^22^.

A standardized consult form was used to guide smoking cessation consultation and to document the consultations. At the initial smoking cessation consultation, the demographic characteristics, plus smoking related and medical history were documented. A carbon monoxide (CO) sample and FEV_1_ measurement was taken and ‘lung age’ calculated and presented to patients. Each participant worked with a pulmonologist, trained in the 2017 evidence-based TOB-G smoking cessation guidelines, to develop a quit plan based on their individual smoking and personal history. A quit date was set in the next 7-14 days with all patients. The clinician provided patients with counselling (i.e. strategies for addressing motivation, cravings and withdrawal in preparation for their quit date) and reviewed personal triggers for tobacco use with the patients. The study intervention was counselling-based and while participants had the option to utilize first line pharmacotherapy (i.e. nicotine replacement therapy, bupropion, or varenicline) it was not provided by the study. Three follow-up sessions were delivered via telephone at 1, 2 and 6 months. For those patients who agreed to attend the in-person visit at the hospital, a CO evaluation was performed at 6 months. At each of the follow-up sessions, smoking status was assessed and the severity of cravings, withdrawal and risk of relapse were assessed. Patients were provided with brief counselling and asked to identify any risk of smoking relapse and to discuss strategies for addressing these high-risk situations.

### Measures

#### Smoking abstinence

The primary outcome measure was self-reported point prevalence smoking abstinence assessed using the Russell Standard^36^. Patient smoking status was assessed at baseline, at 1 (±2 weeks), 2 (±2 weeks) and 6 months (±2 weeks). Point prevalence abstinence was defined as not having smoked in the previous 24 hours at each of the time points assessed. Continuous abstinence (weeks 1-26), defined as not having smoked at the 1-, 2- and 6-month follow-up was also reported. At the 6-month follow-up patients reporting they have quit smoking were asked to provide validation of smoking status using exhaled CO testing (cut-off level 10 ppm).

#### Demographic and smoking related characteristics

Demographic characteristics (age, gender) and medical history of patients were collected at baseline. Smoking history assessed pack-years (years of tobacco use x number of cigarettes per day), actual number of cigarettes per day, and years of smoking. The Fagerström Test for Nicotine Addiction (FTND) was used to assess level of nicotine addiction^26^. The FTND consists of 6 questions scored on a scale of 1-10. A score 0-3 indicates no or low tobacco dependence, a score of 4-6 indicates moderate tobacco dependence, and a score of 7-10 is categorized as high tobacco dependence.

#### Analysis

Descriptive analysis was used to summarize the demographic characteristics of the sample. The proportion of participants who quit smoking was calculated for each follow-up time point. For any patients with missing data we assumed they had returned to active smoking as per Russell Standard^27^. Deceased patients (n=2) were removed from the analysis. SPSS version 24.0 was used to analyse all data.

## RESULTS

### Characteristics of patients

Table 2 summarizes the demographic characteristics of the participants (74% male; age mean±SD = 60.2±7.8). Participants smoked for an average of 29.3±13.8 years or the equivalent of 32.9±19.2 pack-years. At the time of the baseline assessment tobacco users reported smoking an average of 11.7±9.9 cigarettes per day, with the majority (68.0%) of participants reporting they smoked less than 15 cigarettes per day. In all, 18% of participants reported high levels of nicotine addiction as assessed by the FTND. COPD severity ranged from stage I to stage IV, as per GOLD criteria, with a majority of patients in stages II and III (moderate-severe forms), while ‘lung age’ estimates reflected disease severity, accordingly. Among smokers with severe COPD (Stage III, FEV_1_ 30-50%), nicotine dependence as assessed by the FTND was consistently less than 7 (i.e. mild to moderate nicotine addiction). When we evaluated this parameter in this subgroup of Stage III patients, it was revealed that all these patients had the desire to smoke the first cigarette after awaking, much earlier than they could effectively manage to smoke. Patients reported that difficulty in breathing as their disease progressed and other severe COPD morning symptoms resulted in smoking fewer cigarettes per day and smoking their first cigarette later in the day than when they would have otherwise done so.

**Table 2:** Characteristics of COPD pilot study participants, Iasi Romania 2017

*Variable*	*n (%) n=50*
**Gender**	
**Male, n (%)**	37 (74.0)
**Age, n (%)**	
40-49	5 (10.0)
50-59	16 (32.0)
60-69	23 (46.0)
70	6 (12.0)
**FEV_1_, n (%)**	
<30%	10 (20.0)
30-49%	16 (32.0)
50-79%	22 (44.0)
>80%	2 (4.0)
**Lung age, n (%)**	
50-100	19 (38.0)
100-150	22 (44.0)
150-200	5 (10.0)
>200	4 (8.0)
**Cigarettes/day, n (%)**	
01-May	19 (38.0)
Jun-15	15 (30.0)
16-25	13 (26.0)
25	3 (6.0)
**Years of smoking, n (%)**	
0-19	13 (26.0)
20-29	11 (22.0)
30	26 (52.0)
Pack-years, mean (SD)*	32.9 (19.2)
**FTND Score, n (%)**	
Low	21 (42.0)
Moderate	20 (40.0)
High	9 (18.0)

NRT: nicotine replacement therapy, SD: standard deviation

### Smoking cessation

Self-reported rates of smoking abstinence were 30.6%, 44.9% and 64,6% at the 1-, 2-, and 6-month follow-up, respectively (Table 3). Continuous abstinence (weeks 1-26) was 25.0%. Biochemically validated smoking abstinence was documented for 33.3% at the 6-month follow-up. A significant proportion (48.4%) of the sample that self reported abstinence did not complete the CO testing to validate smoking status and as such were classified as tobacco users.

**Table 3:** Rates of smoking abstinence among COPD patients exposed to intervention program

*Variable*	*n/N*	*%*

*Point Prevalence Abstinence*		
**1-month**	15/49	30.6
**2-month**	22/49	44.9
**6-month**		
Self-reported	31/48	64.6
Biochemically verified	16/48	33.3
**Continuous Abstinence (week 4-26)**		
**6-month**	Dec-48	25

## DISCUSSION

Within the context of this pilot study we found the implementation of the 2017 TOB-G tobacco treatment guideline recommendations within European clinical practice was associated with clinically important rates of point prevalence and continuous smoking abstinence among COPD patients sampled. Guideline recommendations were for the most part feasible to implement in clinical practice and acceptable to patients. We achieved high rates (100%) of follow-up contact with patients, providing evidence of the feasibility of the intervention. The pilot intervention integrated spirometry and ‘lung age’ as adjunct treatment strategies, perceived by clinicians involved in the study as a more feasible and tailored way to recommend smoking cessation among COPD, consistent with previous reports^25^. Disease severity and COPD progression risk resulting from previous or continued smoking should be used as principal arguments to motivate cessation in COPD patients who smoke, as motivation and willingness to quit are important parameters of smoking cessation^28^.

Clinical practice guidelines can have an important role in influencing professional practice and guiding the clinical treatment. These guidelines however are infrequently tested in terms of their feasibility, utility and efficacy in ‘real world’ clinical practice settings. Available guidelines for the management of COPD do not offer sufficient attention to evidence-based practices for addressing tobacco use in this important population of tobacco users^29-30^. The TOB-G guidelines were developed to serve as a summary of the latest evidence-based practices for addressing tobacco use^22^. The guideline recommendations were informed by the available literature, clinical experience and vetted by a committee of experts to ensure their applicability to real world clinical settings.

Others have also reported on the value of evidence-based guidelines in influencing tobacco treatment outcomes in clinical settings. Two evaluations of the United States Guidelines for Treating Tobacco Use and Dependence have been published and reported favourable outcomes in terms of efficacy in increasing cessation outcomes among the general population of tobacco users in primary care settings in both a pre-post evaluation and randomized controlled trial design^31-32^. The quit rates documented in this pilot study are somewhat higher than those reported by other investigations of COPD patients that have ranged from 4-34% at 6 months or at a longer follow-up^33^. A recent Cochrane Review examining smoking cessation for people with COPD found evidence that that a combination of behavioural treatment and pharmacotherapy is effective in helping smokers with COPD to quit smoking, but no evidence for any particular form of behavioural counselling or pharmacotherapy^33^. Authors did report some evidence that high-intensity behavioral treatment increased smoking abstinence compared with usual care (Risk Ration, RR=25.38; 95% CI: 8.03-80.22) or low-intensity behavioral treatment (RR=2.18; 95% CI: 1.05-4.49)^33^. The present pilot study was primarily counseling based and included a total of 4 contacts. Higher quit rates may have been documented in the present study if pharmacotherapy was provided to participants. Despite this fact, overall quit rates documented are relatively high and offer preliminary evidence to support the ability of interventions tailored to COPD patients that are based on the TOB-G clinical practice recommendations to support cessation in this high-risk population of tobacco users. Additional research would be required to validate the findings of this initial pilot study.

Our study also found that patients with severe COPD were deterred from smoking in the morning and smoked fewer cigarettes per day as a result of their COPD-related symptoms. Given that time of first cigarette in the morning and number of cigarettes smoked per day are two key questions used by the FTND for assessing nicotine dependence, these suggest that this group of patients may have a much higher dependence to nicotine than the group we registered with the FTND. We hypothesize that the FTND’s reliability may not be as useful in assessing nicotine dependence for this category of patients, and more tailored smoking cessation approaches may be needed to accurately assess patients with severe COPD. This important practical learning from the TOB-G COPD pilot study warrants further examination.

Limitations of our study include the use of a single group non-randomized design. The lack of a control group means that we are unable to confirm a causal relationship between the intervention and changes in smoking outcomes, and it is possible that some participants would have quit smoking without the support of the intervention program tested. Available literature suggests that the natural quit rate among COPD patients involved in clinical studies ranges between 2-9%, indicating that the intervention program was associated with significantly higher rates of smoking abstinence than expected for an unaided quit attempt^33-34^. A significant proportion of the sample did not complete biochemical validation of smoking abstinence and were classified as smokers, so it is possible that these participants had quit but were unable or not interested in traveling to the hospital to provide validation. Other authors have reported on the challenges of compliance with biochemical validation in cessation studies^27^. Given that this was a single centre pilot study, future studies should examine the generalizability of the study findings to other settings and for larger samples of COPD patients. Finally, our study focused on the feasibility and effectiveness of the guidelines on COPD patients who are ready to quit smoking. We did not examine interventions to increase motivation to quit among patients not ready to quit, and we acknowledge that this is an important target for addressing tobacco use among the population of COPD patients^35^.

## CONCLUSIONS

The TOB-G tobacco treatment guidelines for COPD patients were feasible to implement in a ‘real world’ clinical practice setting in Europe and implementation was associated with high rates of patient smoking abstinence compared to that reported in the literature for COPD patients who smoke. The pilot study results will be used to further refine the guidelines to assist with maximizing uptake in clinical settings.
